# Deciphering targeting rules of splicing modulator compounds: case of TG003

**DOI:** 10.1186/s12867-015-0044-6

**Published:** 2015-09-24

**Authors:** Maki Sakuma, Kei Iida, Masatoshi Hagiwara

**Affiliations:** Department of Anatomy and Developmental Biology, Kyoto University Graduate School of Medicine, Konoecho Yoshida Sakyo-ku, Kyoto, 606-8501 Japan; Medical Research Support Center, Kyoto University Graduate School of Medicine, Konoecho Yoshida Sakyo-ku, Kyoto, 606-8501 Japan

**Keywords:** Comparative pharmacogenomics, Comparative transcriptomics, Virtual massive mutagenesis, RNA-targeting therapy, Personalized medicine, Splicing modulator, Polypyrimidine tract, *Duchenne* muscular dystrophy

## Abstract

**Background:**

Recent advances in the development of small chemical compounds that can modulate RNA splicing brought excitement to the field of splicing-targeting therapy. Splicing-targeting therapy tries to ameliorate the disease by altering the exon combination of transcripts to reduce the undesired effect of genetic mutations. However, the knowledge and tools to understand factors contributing to splicing modulator compound sensitivity have been lacking. Our goal was to establish a method to characterize sequence features found in compound sensitive exons.

**Results:**

Here we developed a comparative transcriptomic approach to explore features that make an exon sensitive to a chemical compound. In this study, we chose TG003, a potential drug for *Duchenne* muscular dystrophy, and performed RNA-sequencing on samples from human and mouse skeletal muscle cells, with and without TG003 treatments. We compared TG003 responsiveness between homologous exon pairs and identified 21 pairs in which human exons were skip-enhanced but not mouse exons. We compared the sequence features; splice site scores, number of splicing factor binding sites, and properties of branch sequence and polypyrimidine tracts, and found that polypyrimidine tracts were stronger (longer stretches and richer content of consecutive polypyrimidine) in the mouse TG003 insensitive exons. We also compared the features between TG003 skip-enhanced and insensitive exons within the species, and discovered that human TG003 skip-enhanced exons were shorter and had less splicing factor binding sites than the group of human TG003 insensitive exons. Mouse insensitive exons homologous to human TG003 skip-enhanced exons shared these properties. Our results suggested that these features are prerequisites for TG003 skip-enhanced exons and weak polypyrimidine tracts are defining features, which were supported by a decision tree analysis on all cassette exons in human.

**Conclusions:**

In this study we established a comparative transcriptomic approach, which shed lights on how small chemical compounds modulate RNA splicing. The results described here was the first attempt to decipher the targeting rules of a splicing modulator compound. We expect that this approach would contribute to the precise understanding of the mechanism of TG003-induced splicing modulation, expand target diseases of splicing modulators in general, as well as the development of new splicing modulators.

**Electronic supplementary material:**

The online version of this article (doi:10.1186/s12867-015-0044-6) contains supplementary material, which is available to authorized users.

## Background

Mammalian gene expression requires the accurate excision of introns and ligation of exons from the pre-mRNA by splicing, and approximately 95 % multi-exon genes undergo alternative splicing in human [1]. Alternative splicing contributes to proteomic diversity and organismal complexity because isoforms can have different functions or have non-functional forms to fine-tune the regulation and expression levels of one gene product.

Splicing has been a target of therapy for diseases [2–4]. There are genetic diseases with mutations located near splice sites that cause abnormal splicing such as familial dysautonomia. In this case, a mutation occurred 6 base downstream from exon 20 of IKBKAP gene inhibits inclusion of the exon. Attempts have been made to increase the inclusion of the exon by chemical compounds such as kinetin and RECTAS [5, 6]. There are also diseases that may not have mutations at splice sites, but can be cured by interfering with the splicing process. For example, in order to compensate for the loss of the functional SMN1 gene, the therapy of Spinal Muscular Atrophy intends to increase the expression of SMN2 gene by enhancing the inclusion of a normally skipped exon 7, which is necessary to produce a functional transcript of SMN2 gene [7]. Another example is one of the therapy strategies of *Duchenne* muscular dystrophy (DMD), which is to induce the skipping of exons mutated to be poison exons in the dystrophin gene [8]. Other possible target disorders of this exon-skipping strategy include pseudo-exon diseases [9], which are diseases caused by an emergence of an exon in the intronic regions due to genetic mutations that create a de novo splice site.

Our group developed TG003, a specific CLK (cdc2-like kinase) family inhibitor (CLK1, 2, 4) [10], and identified that TG003 was able to increase the skipping of a mutated exon 31 of the dystrophin gene, and increased the expression of this gene at the protein level [11]. This study opened the possibility of treatment of DMD with TG003, and we further identified a patient whose mutated exon 27 can be enhanced skipping by TG003, whereas none of the wild type exons are affected by TG003 [11]. Advance in knowledge of the features present in TG003 sensitive exons would be very useful for application in personalized subscription of splicing modulators, but this has been obstructed by the intricate mechanism of splicing and the fact that TG003 targets RNA indirectly. The direct targets of TG003 are CLKs, which phosphorylate SR proteins [12–15]. They have different RNA target sequences [16, 17], and the precise rules of how phosphorylation and dephosphorylation of multiple SR proteins affect splice site selection has not been clarified yet [18–20].

In this study we set out to find a rule that can help us understand which exons will be affected by TG003 treatment. Recently, Barbosa-Morais et al. [21] suggested that the outcome of splicing events is determined more by the cis-elements (sequence) than the trans-environment (the set of RNA binding factors in the cell), performing cross-species experiments with human and mouse. This prompted us to design a comparative transcriptome analysis of human and mouse to identify sequence features that make exons responsive to TG003. Comparative studies in human and mouse produced many fruitful results in splicing [22, 23] suggesting that these genomes are similar enough, but at the same time we know that there are many sequence variations. We focused on this difference between the genomes to understand what kind of variations in sequence would gain or lose the response to TG003, assuming that the responses to an artificial product by different organisms are probably not conserved.

In this study we performed RNA-sequencing (RNA-seq) to evaluate the response to TG003 in human and murine skeletal muscle cells. We observed that the general response to TG003 in terms of direction and scope in human and mouse is similar, likely to reflect the fact that the trans-factors that TG003 interferes with are conserved. However we found multiple exons with high sequence similarity but different response to TG003 between human and mouse. Assuming that the difference in response may originate from slight differences in the splicing cis-elements, we proceeded to characterizing sequence features, and found that TG003-sensitive exons are short, have few splicing factor binding sites and have weak polypyrimidine tracts.

## Results

### Validation of RNA-seq data

**Fig. 1:**
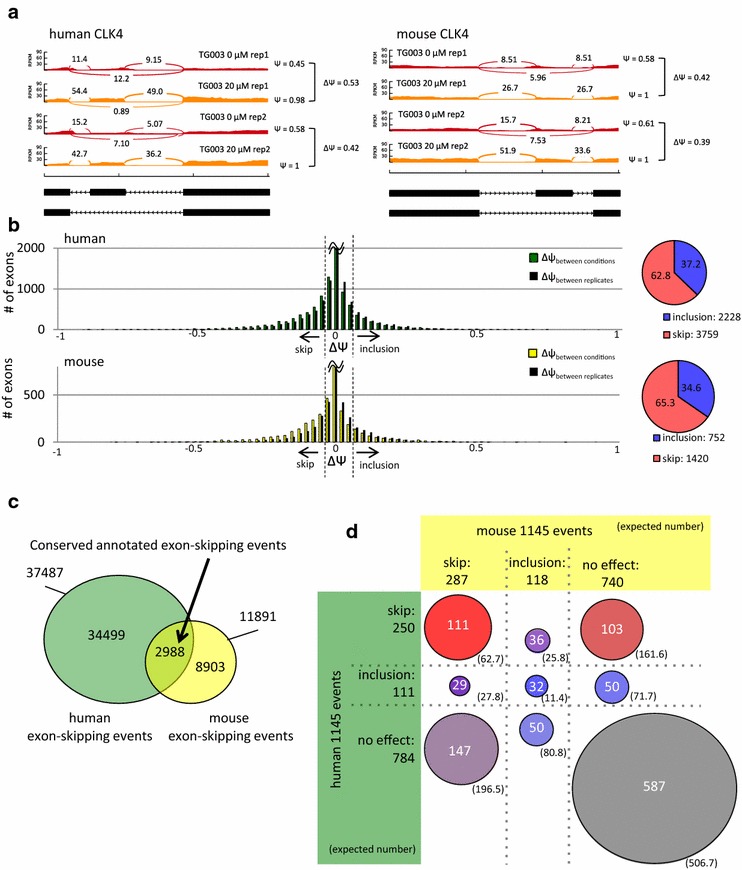
TG003 is a general skip enhancer both in human and mouse. **a** RNA-seq data identifies splicing changes in formerly known TG003 sensitive exons. RNA-seq data was inputted to sashimi-plot for graphical representations. Numbers of junction reads calculated by our method are shown at the junction position. The *black boxes* below represent the exons and the *arrows* indicate the direction of transcription. ψ and Δψ values are calculated on the *right*. **b** *Left* histogram of the average Δψ values [Δψ_avg_ = (Ψ_DMSO.rep1_ − Ψ_TG003.rep1_ + Ψ_TG003.rep2_ − Ψ_DMSO.rep2_)/2)] of all annotated exon-skipping exons are shown in *green* for human and *yellow* for mouse. The distribution of difference in ψ between replicates of the same condition are shown in *black* to show the average baseline fluctuation of ψ values [(Ψ_DMSO.rep1_ − Ψ_DMSO.rep2_ + Ψ_TG003.rep1_ − Ψ_TG003.rep2_)/2]. The *black dashed line* shows the cutoff value of Δψ = −0.05 for skip and Δψ = 0.05 for inclusion. *Right* pie chart of the ratio of skip-enhanced (Δψ_avg_ ≤ −0.05) and inclusion-enhanced (Δψ_avg_ ≥ 0.05) exons. **c** Venn diagram of the overlap between the number of human and mouse annotated exon-skipping events in Ensembl. **d** Distribution of 1145 expressed and consistent conserved annotated exon-skipping events in both species into the nine categories. *Circles* roughly show the scales, and the *numbers* show the exact counts. *Numbers in the brackets* show the expected values for each categories under the assumption of no-conservation of TG003 effects between the species

In order to investigate the TG003 sensitive exons in the two species, we performed RNA-seq on human skeletal muscle cells (hSkMC) and mouse C2C12 cells treated with 20 μM of TG003 or no treatment (0.4 % DMSO) for 4 h. The numbers of mapped reads and mapping rates are summarized in Additional file 1: Table S1. To quantify the change in splicing, we calculated Δψ, which stands for difference (Δ) in Percent Spliced In (ψ), and measures the difference in the inclusion rates (ψ) between the two conditions (see methods). In order to confirm the effect of TG003, we looked at CLK1 and CLK4 exon 4, which we have consistently observed response to TG003 in different types of cells [10, 15]. RNA-seq reproduced the effect of TG003 in CLKs in our samples as did RT-PCR (Fig. 1a drawn in sashimi-plot [24], Additional file 2: Figure S1).

### TG003 is a splicing modulator with an orientation to skip enhancement both in human and mouse

Next we tried to characterize the effect of TG003 to alternative exon-skipping events in general. We calculated the Δψ values for all 37287 and 11891 annotated alternative exon-skipping events in human and mouse Ensembl database, respectively. We observed a slight negative shift in the distribution of Δψ values compared to the baseline fluctuation of Δψ values (Δψ between replicates), both in human and mouse, indicating that TG003 is a general skip enhancer, and the ratio of skip-enhanced (Δψ ≤ −0.05) to inclusion-enhanced (Δψ ≥ 0.05) was remarkably similar between human and mouse (Fig. 1b).

We next investigated the conservation of response to TG003 at the level of individual alternative exon-skipping events. To determine this we made a subset of 2988 sequence-conserved exon-skipping events from all the annotated exon-skipping events in human and mouse (Fig. 1c). These events had on average 93.6 % sequence similarity over 98.5 % of the exonic region. We took 1995 events that had sufficient expression in both species and filtered out events that had inconsistent TG003 effects between two replicates in either of the species, which left us with 1145 events. For each event, we classified events into skip (Δψ ≤ −0.05), inclusion (Δψ ≥ 0.05) and no effect (−0.05 < Δψ < 0.05) for human and mouse respectively, and grouped each event into one of the nine categories as shown in Fig. 1d. The number of events in the categories that have exons with conserved response to TG003; “human-skip mouse-skip”, “human-inclusion mouse-inclusion”, and “human-no effect mouse-no effect”, were higher than expectation values which were calculated under the assumption that there is no conservation of TG003 effects. The differences between observed values and expectation values were significant with Chi square test (p < 2.2e−16).

### Virtual massive mutagenesis mimicked by species-comparing transcriptomic analysis

The previous results showed that the general response to TG003 is conserved, possibly reflecting the fact that the trans-factors that TG003 works through is generally conserved, but there were several exons that did not respond in the same manner (Fig. 1d). In order to find the rules that make an exon responsive to TG003, these pairs of exons would be most informative because they respond differently though there is only small differences in the sequence.

**Fig. 2:**
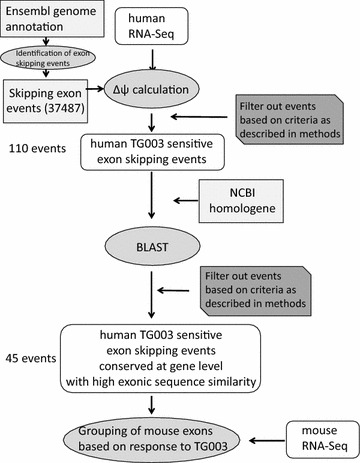
Schematic of species-comparing transcriptome analysis. Flow chart of the process determining human TG003 skip-enhanced exons and its counterpart mouse homologous exons. *Squares* represent public annotations, *rounded squares* represent our RNA-seq data, *oval circles* represent manipulations. *Dark gray squares* with cut sides show that there was a filtering process. *Numbers on the side* indicate how many events were left after the manipulations

**Fig. 3:**
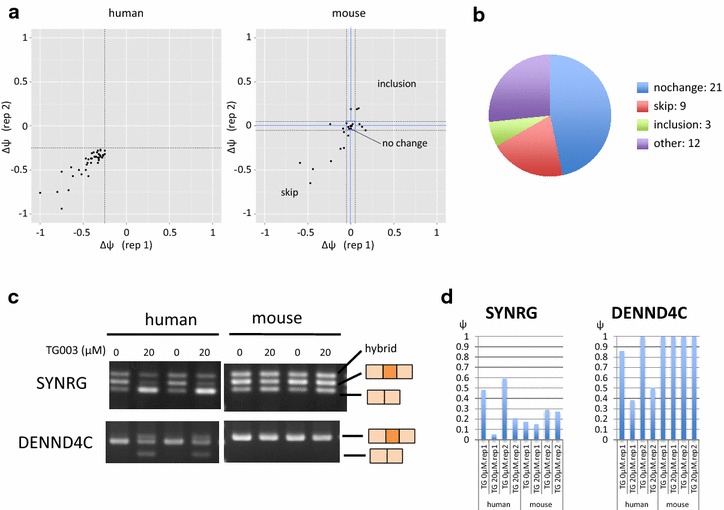
Distribution of Δψ values of human TG003 skip enhanced exons and mouse counterpart homologous exons. **a** Scatter plot of the Δψ values in the two replicates of human TG003 skip-enhanced exons (*left*) and their counterpart mouse homologous exons (*right*). **b** Pie chart of the grouping of mouse counterpart homologous exons; skip (Δψ ≤ −0.05 in either of the replicates and Δψ < 0 in the other), no change (−0.05 < Δψ < 0.05 in both replicates), inclusion (Δψ ≥ 0.05 in either of the replicates and Δψ > 0 in the other), and others. Others include: no expression (Δψ not calculated because there was no junction read), inconsistent [(Δψ ≥ 0.05 and Δ ψ ≤ 0) or (Δψ ≤ −0.05 and Δψ ≥ 0)] occur at the same time in the two replicates, psi too low (ψ < 0.05), which never provide Δψ ≥ 0.05. **c** Experimental validation by RT-PCR of the two representative cases of the 21 pairs, that is, SYNRG (Ensembl: ENST00000585472.E20, Ensembl: ENSMUST00000183456.E22) DENND4C (Ensembl: ENST00000494124.E27, Ensembl: ENSMUST00000142837.E30). **d** The ψ values from our RNA-seq data are shown in bar graphs for the two selected genes

We focused our attention on human skip-enhanced splicing events and expanded our comparison set to all possible internal mouse exons. The schematic of the analysis is shown in Fig. 2. We first constructed a set of human skip-enhanced splicing event set with stricter criteria. We ensured that the level of gene and junction expression are sufficiently high, and obtained splicing events with Δψ ≤ −0.25 that also passed *t* test with p value <0.05. This left us with 110 human TG003 skip-enhanced splicing events as our starting set. We searched for homologous exons in all internal mouse exons, and obtained 45 human skip-enhanced splicing events with mouse homologous exons. We calculated the Δψ values for the mouse internal exons (they included annotated and unannotated exon-skipping event), and the distribution of the Δψ values of these pairs of splicing events are shown in Fig. 3a (Ensembl IDs and Δψ values are summarized in Additional file 3: Table S2). We grouped the mouse splicing events depending on their response to TG003 as shown in Fig. 3b. We confirmed the RNA-seq data of the representative cases by RT-PCR (Fig. 3c), which reproduced the response to TG003 as was observed by the RNA-seq data (Fig. 3d). We also confirmed representative cases by RT-PCR from the group of exons which were skip-enhanced in both species (Fig. 3b, red fraction) by TG003 as well as the group of exons which were skip-enhanced only in the mouse events identified in a separate analysis (Additional file 4: Figure S2).

**Fig. 4:**
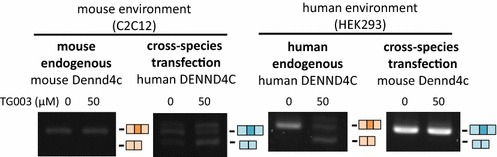
Difference in response to TG003 is derived from difference in sequence and not trans-environment. *Left* RT-PCR of endogenous mouse Dennd4c exons 28–31 and transfected human DENND4C exons 26–29 in pEGFPc1-human-DENND4c-e29–31 transfected C2C12 cells. *Right* RT-PCR of endogenous human DENND4C exons 25–29 and transfected mouse Dennd4c exons 29–31 in pEGFPc1-human-DENND4c-e29–31 HEK293 cells. After transfection both experiments were treated with 50 µM of TG003 for 4 h before harvest

In order to examine our hypothesis that the differential results were obtained due to changes in cis-element, and not trans-environment we selected DENND4C from the group and cloned the human DENND4C (Ensembl: ENST00000494124) exon 26–28 and mouse Dennd4c (Ensembl: ENSMUST00000142837) exon 29–31, and cross-species transfected the minigene reporter. The results showed that human DENND4c exon 27 were skip-enhanced even in the mouse trans-environment of C2C12 cells, when the mouse endogenous Dennd4c did not respond to TG003 (Fig. 4, right). Mouse Dennd4c exon 30 did not respond to TG003 in the human trans-environment of HEK293 cells, when the human endogenous DENND4C exon 30 were skip-enhanced by the treatment of TG003 (Fig. 4, left). These results suggested that the difference in response to TG003 originates in the difference in sequences.

### Polypyrimidine tract tends to be weaker in TG003 skip-enhanced exons

We then tried to identify the sequence features that determines whether the exon responds to TG003. We constructed a set of all homologous pairs of exons that were both insensitive to TG003 as a control, and did the following comparisons. (A) Comparison between human TG003 skip-enhanced exons and mouse homologous TG003 insensitive exons that were identified in the previous section. (B) Comparison between the control set of homologous human and mouse TG003 insensitive exons if there were any significant differences in comparison A. (C) Comparison between human skip-enhanced exons used in A and human insensitive exons used in B. (D) Comparison between mouse insensitive exons used in A and mouse insensitive exons used in B. We expected that the four comparisons together would reveal the true determinants of TG003 response. We quantified the following features for every exon; exon length, number of ESEs (exonic splicing enhancers), number of ESSs (exonic splicing silencers), strength of 5′ splice site and 3′ splice site (branch sequence, polypyrimidine tract, 3′ splice site), intron length, 5′ splice site of upstream introns and 3′ splice site of downstream introns, which are known as contributing factors for the outcome of splicing [25–28].

**Fig. 5:**
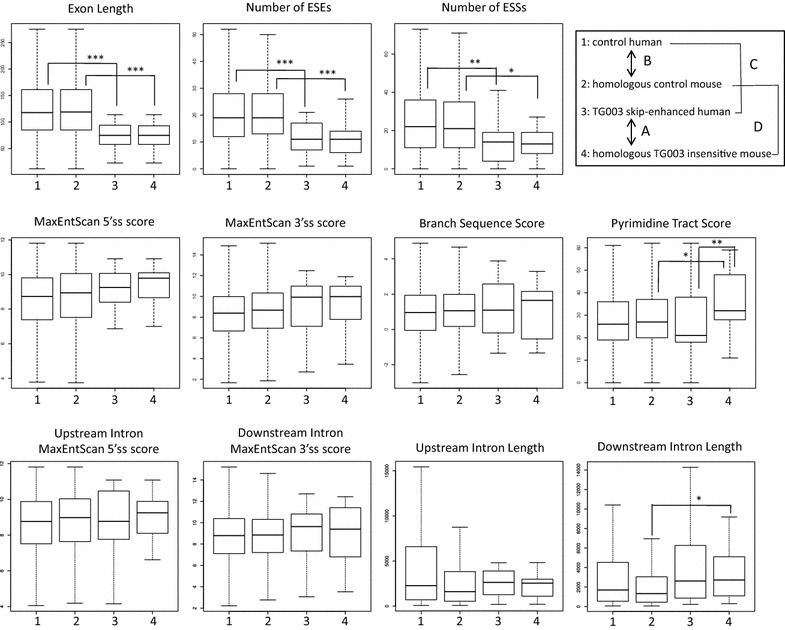
Sequence features comparisons between different exon sets identify characteristics of TG003 skip-enhanced exons. Box plots of scores of sequence features of control homologous exon pairs (n = 2335) and human TG003 skip-enhanced and their insensitive mouse counterpart exon pairs (n = 21). Median is shown by *bold horizontal line* and *boxes* are drawn between the third quartile (*top of box*) and first quartile (*bottom of box*). Outliers are either three times interquartile range or more above the third quartile, or three times interquartile range or more below the first quartile, and are not shown in the plot. The maximum and minimum are shown with the whiskers. (*) indicates p value <0.05, and (**) p value <0.01, (***) p value <0.001, calculated by Wilcoxon rank sum test

Figure 5 shows the distribution of scores of the four groups of exons in box plots, and values used are summarized in Additional file 5: Table S3, Additional file 6: Table S4, and graphical representations of upstream introns are shown in Additional file 7: Figure S3. There were two notable types of features. One type of the feature is that the feature scores are significantly different in both comparison C and D; found in the comparisons of exon length, number of ESEs, number of ESSs. The other is that the feature scores are significantly different in comparison A but not in B; found in the comparison of polypyrimidine tract score.

As to the first type of features, the results from comparison C suggest that short exon length, few number of ESEs and few number of ESSs are important prerequisites for exons to be responsive to TG003, but the results from comparison D show that the features are present in TG003 insensitive exons, suggesting that these features are not sufficient for an exon to be responsive to TG003.

The second type of feature, which showed significant difference in comparison A is what seemed to be the determinant of TG003 response, that is, “weak” polypyrimidine tract. The fact that there was no significant difference from comparison B suggests that this difference is not due to species difference. However the fact that there was no significant difference in comparison C suggests that weak polypyrimidine tract is not a determining factor in every exon, but likely becomes a determining factor in exons which are short and have few splicing factor binding. We also observed that the insensitive mouse exons that are homologous to human exons have significantly stronger polypyrimidine tract (comparison D), which seems to suggest that short exons with few splicing binding sites are likely targets of TG003 as long as the polypyrimidine tract is not too strong.

In addition, we noticed that downstream introns were significantly longer in TG003 insensitive exons that were homologous to human TG003 skip-enhanced exons than in the control TG003 insensitive exons (comparison D). However, there was no tendency for human TG003 skip-enhanced exons to be shorter than human control TG003 insensitive exons (comparison C).

Box plots of homologous exon pairs that are both skip-enhanced by TG003 show similar tendency, and importantly polypyrimidine tract is not strong compared to those of their human homologous counterpart exons (Additional file 8: Figure S4).

### Skip-enhanced exons are enriched within the groups of exons that have weak polypyrimidine tracts and few splicing factor bindings

**Fig. 6:**
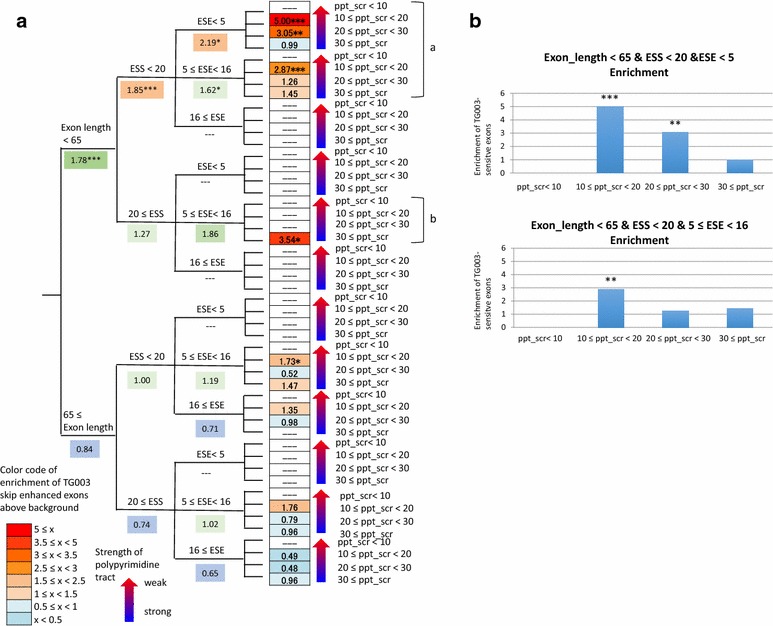
Groups with weak polypyrimidine tracts are more enriched with TG003 skip-enhanced exons. **a** Decision tree model of TG003 skip-enhanced exons. *Boxes* are colored according to their enrichment level with the *color code* shown at the bottom. **b** Bar graph of enrichment in the four groups of polypyrimidine tract strength in exons with short (length <65 nt), few splicing factor binding (ESE < 5 or 5 ≤ ESE < 16, ESS < 20). (*) indicates p value <0.05, and (**) p value <0.01, (***) p value <0.001, calculated by hypergeometric test

In the previous section we observed that human exons that are skipped by TG003 are short and have few number of splicing factor binding, and among those, we found that polypyrimidine tract strength is critical in the decision by the splicing machinery whether to skip the exon or not. In order to confirm that this trend holds for human exons that are not necessarily homologous to mouse exons, we focused on all 20897 human annotated exon-skipping events that were sufficiently expressed and assigned sequence feature scores. We grouped exons depending on the length, number of positive and negative splicing factor binding sites, and polypyrimidine tract score, and observed the distribution of the 110 skip-enhanced events (Δψ ≤ −0.25) by TG003 that we defined previously (Fig. 2). The detailed results are summarized in Additional file 9: Table S5. In the groups of short exons with few splicing factor binding sites, we observed a clear trend that groups of exons with weaker polypyrimidine tract are more enriched with TG003 skip-enhanced exons (region a in Fig. 6a). Especially for the group of “Exon length < 65, ESS < 20, ESE < 5”, strong association between low polypyrimidine tract scores and high enrichment of TG003 skip-enhanced exons were observed (Fig. 6b, upper panel). These properties were weakened in a group with exons with relatively large numbers of ESE sites; 5 ≤ ESE < 16 (Fig. 6b, lower panel). These graphs also suggested that too weak polypyrimidine tract (polypyrimidine tract score < 10) makes the exons TG003 insensitive rather than skip-enhanced. We also observed enrichment in groups of exons with strong polypyrimidine tracts in short exons with high number of ESSs (region b in Fig. 6a), which suggests that for exons that do not satisfy all of the characteristics (short exon length AND few ESE AND few ESS), other rules remain to be discovered.

## Discussion

In this study we found some rules that can help us understand which exons will be affected by TG003 by a comparative transcriptomic analysis approach. We showed that TG003 skip-enhanced exons are short exons with few splicing factor binding sites and have weak polypyrimidine tracts. TG003 is a CLK family inhibitor and is known that CLK1 phosphorylates the SR proteins [12–15] and phosphorylated SR proteins are known to enhance exon inclusion by binding to exonic elements [18]. Therefore the fact that TG003 skip-enhanced exons have few ESEs is at first counterintuitive. However recent research [16] suggests that SR proteins work synergistically and competitively, and it is more reasonable that the effect of TG003 is more dramatic on exons that have fewer opportunities for compensatory activity by other factors that enhance exon inclusion.

**Fig. 7:**
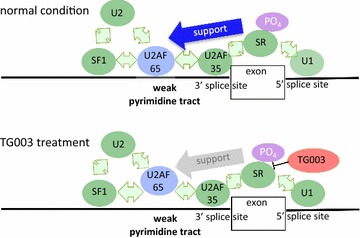
Model of mechanism of TG003 exon skip enhancement. Model of mechanism of TG003 exon skip-enhancement. We showed that TG003 skip-enhanced exons have weak polypyrimidine tract, which is known to be bound by U2AF65. Some exons require support from other splicing factors for splicing reaction. Treatment of TG003 might result in exon skipping by the loss of the support

The polypyrimidine tract is rich with pyrimidine nucleotides, especially uracil, located about 5–40 base pairs before the 3′ end of the intron to be spliced, and associates with many protein factors such as the U2 small nuclear RNA auxiliary factor 2 (U2AF65) and polypyrimidine tract-binding protein (PTB). We found that the polypyrimidine tract strength is one of the key determinants of TG003 action, which agrees well with past research that showed splicing enhancers stimulated U2AF65 recruitment to pre-mRNA with weak splice sites [29]. When the polypyrimidine tract is rather weak for the binding of U2AF65, the exon depends more on the splicing factors binding to the ESE, and the inhibition of SR proteins by TG003 results in exon skipping (summarized in Fig. 7). To further explore the possibility that weak U2AF65 binding to the polypyrimidine tract is mediating the exon skipping effect of TG003, we compared splicing changes in U2AF65 knock down (KD) cells and our TG003 treated cells. The RNA-seq data of U2AF65 KD cells was obtained by Shao et al. and was deposited in NCBI SRA with accession number SRR1582594 [28]. We listed commonly expressed genes among the U2AF65 KD dataset and our current dataset, and took the overlap of the skip-enhanced exons of the commonly expressed genes in the two dataset. We obtained 373 exons which were skip-enhanced by both TG003 treatments and U2AF65 KD (Additional file 10: Table S6). This number was almost twice larger than the expected value: 188, which was calculated under the assumption that effects of TG003-treatment and U2AF65 KD were independent. This difference was statistically significant (p < 2.2e−16) with Chi square test. The fact that U2AF65 KD and TG003 treatment enhance skipping of a similar set of exons supports our hypothesis that TG003 affects exon-skipping via changes of interaction between U2AF65 and polypyrimidine tract.

Besides, the mutation found in the Duchenne patient, who was sensitive to TG003, was located in an ESE of exon 31 of *Dystrophin* gene, which has a weak polypyrimidine tract, as we previously reported [11]. When we strengthened the polypyrimidine tract of the mutated exon 31, the skip-enhancing effect of TG003 was weakened (Kataoka et al. unpublished data).

In evolutionary terms, treatment to artificial products such as TG003 is an event that has never occurred in the history of evolution and so it is no surprise that evolution did not prepare different organisms to act in the most fitted way to small chemical compounds. Also the combinatorial nature and functional redundancy of splicing factors may have permitted wild sequence variation through evolution while still preserving the same splicing outcome. This brings an important point to strategies in developing drugs, which is that mouse models and murine cells may not be the best model for splicing drug analysis. Paying attention to intronic sequence as well as the exonic sequences is important even if the default splicing pattern is the same.

Identifying target sequences of chemical compounds that interfere with splicing is difficult. In contrast to RNA-binding proteins, where methods such as HITS-CLIP (High-throughput sequencing of RNA isolated by crosslinking immunoprecipitation) offer a reliable way to identify binding sequences of the protein of interest, there is no tangible way to “clip” out the target sequence of a chemical compound such as TG003 that mildly affects many key splicing regulators. However, our comparative transcriptomic approach successfully identified key factors which determine the sensitivity to TG003. We obtained this result through sequence analysis and did not need to go through extensive characterization of TG003 target proteins. Although more research is needed to precisely predict the target exons of TG003 by sequence inspection, our method proved to bring insight into the features of target sequence of compounds without prior knowledge of the target proteins of the compound.

## Conclusions

We used a comparative transcriptomics analysis approach to identify sequence features which lead exons to be skip-enhanced and shed light on the mechanism of TG003 splicing modulation (Fig. 7). Our work contributes to the field of developing splicing drugs by bringing in this method as well as by calling attention to the fact that splicing events of different species respond differently to compounds even if the default splicing pattern is similar and exonic sequence conserved. Our approach is applicable to other splicing modulators and comparison with more than two species may enable examining even finer details of the target sequence features. Learning the target sequences of each splicing drug is useful since many patients who have mutations that can benefit from splice-modulation do not have the same mutation. For example, Nishida et al. [11] identified two patients of DMD whom may benefit from TG003, and these two patients had different mutations in different exons, and there may be more patients who we do not know yet that TG003 may be able to help. Characterization of the target sequences of multiple splicing modulators would enable offering customized splicing drugs to patients whose desired splicing modulations may differ in each case.

## Methods

### Cell culture and plasmid transfection

Human skeletal muscle cells (hSkMC) and C2C12 myoblast cells were maintained in DMEM, supplemented with 10 % FBS. Both cells were differentiated at around 80 % confluence by switching medium to DMEM supplemented with 0.5 % FBS. They were allowed to differentiate for 10 days, by which point fusion of myoblasts with multinucleated syncytia were observed. Then cells were treated with either 0.04 % DMSO or 20 µM TG003 for 4 h.

For plasmid transfection we used undifferentiated C2C12, HEK293 cells maintained in DMEM, supplemented with 10 % FBS. HSkMCs were not used due to low transfection efficiency. 2 µg of plasmids were transfected with Lipofectamine 2000 to cells cultured in 6-well dishes following standard procedures. Cells were treated with DMSO or 50 µM of TG003 4 h before harvest.

### Plasmid constructions

To construct human DENND4C exons 26-29 and mouse Dennd4c exons 29-31 splicing reporter, we PCR amplified the triplet of exons including the flanking introns from Human reference DNA male (Catalog #5190-4370, Lot #0006139685 Agilent) for human reporter and B6 mouse genome for mouse reporter. We used XhoI/BamH1 sites for human amplified segment insertion and BglII/SalI sites for mouse amplified segment insertion to the pEGFPc1 vector (Clonetech). Primers used are summarized in Additional file 11: Table S7. The internal exon and each flanking 100 bp intronic regions were verified by sequencing.

### RNA extraction, RNA-seq library preparation, RT-PCR

Total RNA was extracted from hSkMC and C2C12 cells with Sepasol (Nacalai Tesque). 5 µg of total RNA was poly-A purified by ambion Dynabeads mRNA DIRECT Micro Kit (Life Technologies), and 45 ng of poly-A purified RNA were used for RNA-seq library preparation using Ion Total RNA-seq Kit v2 (Life Technologies). Single-end sequencing was performed on Ion Proton System.

Reverse transcription was done by Prime Script (TaKaRa) with polyT primers, and PCR was done with ExTaq (TaKaRa) following the manufacture’s protocol. PCR conditions were a cycle of 94 °C for 2 min followed by 35 cycles of 98 °C for 10 s, 55 °C for 30 s, and extension time depending on the length of the transcript. The primers used are summarized in Additional file 11: Table S7.

### RNA-seq analysis

In order to ensure read quality, we discarded reads below 100 nt or 17 of average Phred quality score. We used Tophat 2 [30] with options—no-coverage-search—library-type fr-second strand to map the sequenced reads to the Ensembl reference genome with assemblies GRCh37.75 for human and GRCm38.75 for mouse [31]. When calculating junction reads, we filtered out reads which do not extend 8 nt into either of the flanking exons, following the method of Barbosa-Morais et al. [21]. The gene expression levels and junction expression levels were calculated in reads per kilo-base per million reads (RPKM), which is a method to compare the number of reads across different samples. We quantified the reads per kilo-base per 30 million (RPK30 M) to get a better resolution. Exon-skipping events were not used in the analyses if the gene which the event is on has expression less than 16 RPK30M in all of the replicates or the total of consecutive three junction reads less than 4 RPK30M in any of the replicates in at least one of the conditions. Analyses using all mouse internal exon set did not use this expression filter (sequence feature comparison section).

### Retrieval of annotated exon-skipping event

We extracted exon-skipping events from Ensembl annotations [31]. We defined exon-skipping events with the alternative exon and the two flanking introns (Additional file 12: Figure S5A). In the exon-skipping event extraction algorithm from the Ensembl database, for each gene model of a gene we stored the genomic coordinates of the 5′ end of the upstream intron (arrow 1 in the Figure S5A) and the 3′ end of the downstream intron (arrow 4) for each internal exons. Then, if any other gene model has an intron with the 5′ coordinate coinciding with the 5′ end of the upstream intron of the gene model of interest, and 3′ coordinate with the 3′ end of the downstream intron of the gene model of interest (arrows 5, 6), i.e. an intron without any annotated exon, we defined the case as exon-skipping events. The exon-skipping events were described with 4 points of genomic coordinates (arrows 1–4), and cassette exons were described with the coordinates shown with arrows 2 and 3. The events and cassette exons found with this algorithm were called “annotated exon-skipping events” and “annotated cassette exons” in this study, respectively.

### Splicing change assessment and percent inclusion calculation

To assess splicing change, we obtained the expression of the three junctions in triplets of exons and calculated the percent spliced in (PSI, ψ) levels [32] as shown in Additional file 12: Figure S5B. Then in each replicates we subtracted the DMSO ψ values from the TG003 ψ values to obtain the difference in ψ values (Δψ_TG003_treatment_; Δψ = ψ_TG003.rep.n_ − ψ_DMSO.rep.n_). To evaluate the distribution of Δψ values, we calculated the difference between the ψ values of the replicates of the same condition as a reference of baseline fluctuation (Δψ_between_replicates_ = ψ_DMSO.rep.2_ − ψ_DMSO.rep.1_ and ψ_TG003.rep.2_ − ψ_TG003.rep.1_) used in Fig. 1b.

### Homologous exon definition

The homologous exon pairs of the following two sets were defined. One is human annotated cassette exons against mouse annotated cassette exons, and the other is human annotated cassette exons against all mouse internal exons. We obtained the Ensembl IDs for the genes with the human annotated exon-skipping events and converted them to NCBI IDs and obtained the numbered homologene group IDs, which were linked to Gene Symbol [33]. We used homologene ID as a key to link the human gene with the mouse homologous gene. Then for each comparison we used BLAST [34] to align with the annotated cassette exons in the homologous gene of the human exon’s mouse homologous gene for the first set and all exons of the said gene for the second set. After blast (-p blastn) alignment, we selected the homologous exon based on their sequence identity given by BLAST and coverage calculated by dividing the length of the aligned segment by the human and mouse exon length, respectively. We selected exons with percent identity >70 % and human and mouse coverage >0.9, respectively. Then we mapped back the Ensembl IDs to its genomic coordinates and selected mouse homologous exons with the highest percent identity and sum of the two coverages. In this study, we compared not only the homologous cassette exons but also the flanking introns. For human exons we retrieved the flanking exons, which define the flanking introns, specified by the Ensembl IDs we started with. For mouse exons, when BLAST search identified exons which had multiple flanking exons in the annotation we obtained flanking exons information based on the exons defined as homologous with human cassette exons. If Ensembl gene sets contained several sets of flanking exons, we chose the one with highest total junction reads expression across all samples. We note that the same mouse exon is possibly assigned as a homologous exon against multiple human cassette exons, due to exon/gene duplication events during the evolution, or due to alternative 5′ splice sites or 3′ splice sites unique to the human species.

### Sequence feature characterization

We scored six splice signals that are important for exon recognition, which are shown in Additional file 12: Figure S5C. Scoring of 5′ splice site and 3′ splice site was done by inputting 9-mer sequence of 5′ splice site, and 23-mer sequence of 3′ splice site in the software MaxEntScan [35]. Scoring of exonic splicing enhancers and silencers were based on the database SpliceAid [36]. We took all the binding motifs for each splicing factors registered in SpliceAid, and looked for its presence in each exon. Because we searched binding sites within the exonic regions, we treated binding sites with positive scores in the SpliceAid database as ESEs and, ones with negative scores as ESSs. We separately added up the number of ESEs and ESSs for each exon. For the branch sequence scores and polypyrimidine tract scores, we used SVM-BP finder [37]. We used 100 nucleotides of upstream introns as the input and predicted the branch point. We used human scoring system for the analysis of both species in order to avoid score variances for the same sequences in different species. When multiple branch points were predicted, we retrieved the prediction of the highest svm_scr and obtained the polypyrimidine tract score and branch sequence score from that prediction.

The control set was constructed in the following way. We first obtained exons from the annotated alternative exons in human with Δψ = 0 in both replicates, and identified their mouse homologous exons from the set of all mouse internal exons as described in the previous section. Then we selected mouse exons which had Δψ = 0. The size of the sets described in the figure legends. The nonparametric Wilcoxon rank sum test was used in order to compare the human and mouse homologous exon sets.

### Enrichment analysis

We obtained 20,897 expressed annotated exon-skipping event set among the total of 37,497 annotated alternative splicing events. Some events were excluded because SVM-BP could not predict any branch points and no scores were calculated. We grouped the 20,897 exon-skipping events in 48 groups depending on their exon length (0–64 nt, 65 nt), number of ESEs (0–4, 5–15, 16), number of ESSs (1–19, 20), and polypyrimidine tract score (0–9, 10–19, 10–29, 30). We counted the total number of members of each group and the number of the 110 skip-enhanced (Δψ ≤ −0.25) exons categorized into this group. A minimum of 3 strongly skip-enhanced exons per group was required to proceed into the enrichment analysis. We calculated the enrichment by dividing the number of strongly skip-enhanced exons by the total number of members of the group, and then dividing this by 0.00526, the average fraction of strongly skip-enhanced exons in random categorization (110/20,897 = 0.00526). Hypergeometric tests were performed to calculate the enrichment p values.

## Additional files

**Additional file 1: Table S1.** Summary of RNA-seq data. Total mapped reads and mapping rates are indicated for all samples used in this study.
**Additional file 2: Figure S1.** A RNA-seq data visualization of CLK1 exon 4 in both human and mouse.RNA-seq data was visualized by sashimi-plot. Numbers of junction reads shown are calculated by our method. B Experimental validation by RT-PCR.
**Additional file 3: Table S2.** Comparison of Δψ values of 45 TG003 skip-enhanced human exons and their homologous mouse exons. Ensembl IDs and all information used to identify and classify mouse exons that correspond to human skip-enhanced exons (Δψ ≤ 0.05) are provided with in this table.
**Additional file 4: Figure S2.** A Experimental validation of RNA-seq data of different groups.RT-PCR of two exon pairs that are both skip-enhanced by TG003 and one pair of human TG003 skip-enhanced and mouse insensitive exons. B Graphs show the RNA-seq derived ψ values.
**Additional file 5: Table S3.** Exon feature scores of all 21 human TG003 skip-enhanced exons and mouse insensitive exons. Ensembl IDs, genomic coordinates and all information used to draw Figure 5 box plots (column 3 and 4) are provided with in this table.
**Additional file 6: Table S4.** Exon feature scores of all 2335 control exon pairs. Ensembl IDs, genomic coordinates and all information used to draw Figure 5 box plots (column 1 and 2) are provided with in this table. Some SVM-BP scores are not given because SVM-BP did not find any branch point that satisfies the required conditions of a branch point.
**Additional file 7: Figure S3.** Alignment and upstream intron features of all 21 exon pairs. Sequence alignment of 100 nt of upstream introns and branch sequence scores, pyrimidine tract scores, and svmbp scores predicted by SVM-BP are shown. The first entry is an example and shows all the captions.
**Additional file 8: Figure S4.** Boxplots of homologous exon pairs that both respond to TG003. Boxplots with additional data pair set (5-6 both skip-enhanced, n = 9 as in Figure 3B red) to Figure 5. Significance is shown only for Wilcoxon rank sum test performed with 5 or 6. Median is shown by bold horizontal line and boxes are drawn between the third quartile (top of box) and first quartile (bottom of box). Outliers are either 3 times interquartile range or more above the third quartile, or 3 times interquartile range or more below the first quartile and are not shown in the plot. The maximum and minimum are shown with the whiskers.
**Additional file 9: Table S5.** Values used in decision tree analysis.Table to accompany Figure 6.
**Additional file 10: Table S6.** Comparison result of skip-enhanced exons between TG003 treated cells and U2AF65-KD cells.
**Additional file 11: Table S7.** Primers used in this study.
**Additional file 12: Figure S5.** Methods to identify TG003 sensitive exons and sequence features of interest.A. The positions of the genomic coordinates used to identify exon-skipping events are shown in red with numbered arrows. Boxes represent exons and lines represent introns. B. Overview of the calculation of percent spliced in (ψ) values. Red lines represent RNA-seq reads mapped to the genome. Circled numbers represents the number of reads mapped to the exon junction right below it. C. The positions and length of the sequences used as inputs to the respective scoring systems.

## Abbreviations

DMD: *Duchenne* muscular dystrophy
CLK: cdc-like kinase
SR proteins: serine/arginine-rich proteins
RNA-seq: RNA-sequencing
hSkMC: human skeletal muscle cell
ψ (PSI): percent spliced in
ESE: exonic splicing enhancer
ESS: exonic splicing silencer
ppt_scr: polypyrimidine tract score
U2AF65: U2 small nuclear RNA ausiliary factor 2
PTB: polypyrimidine tract-binding protein
RPKM: read per kilo-base per million reads
