# Supplementary figures and images for: Deciphering targeting rules of splicing modulator compounds: case of TG003
Source: BMC Mol Biol. 2015 Sep 24;16:16. doi: 10.1186/s12867-015-0044-6 (PMC4580995; doi:10.1186/s12867-015-0044-6)

A

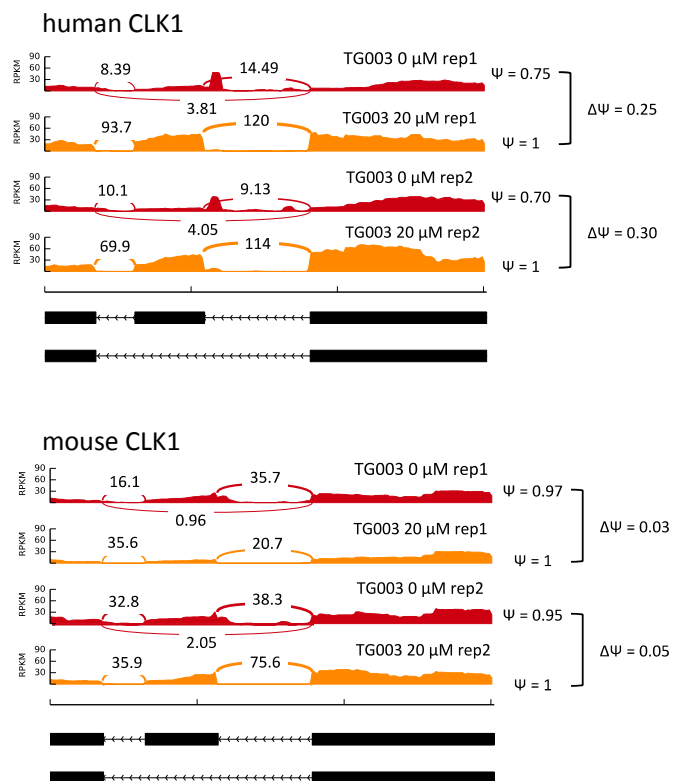

B

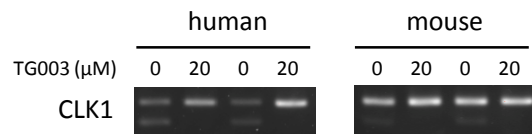

Supplement: Supplementary file 2 — Additional file 2: Figure S1. A RNA-seq data visualization of CLK1 exon 4 in both human and mouse.RNA-seq data was visualized by sashimi-plot. Numbers of junction reads shown are calculated by our method. B Experimental validation by RT-PCR. [file 12867_2015_44_MOESM2_ESM.pdf]

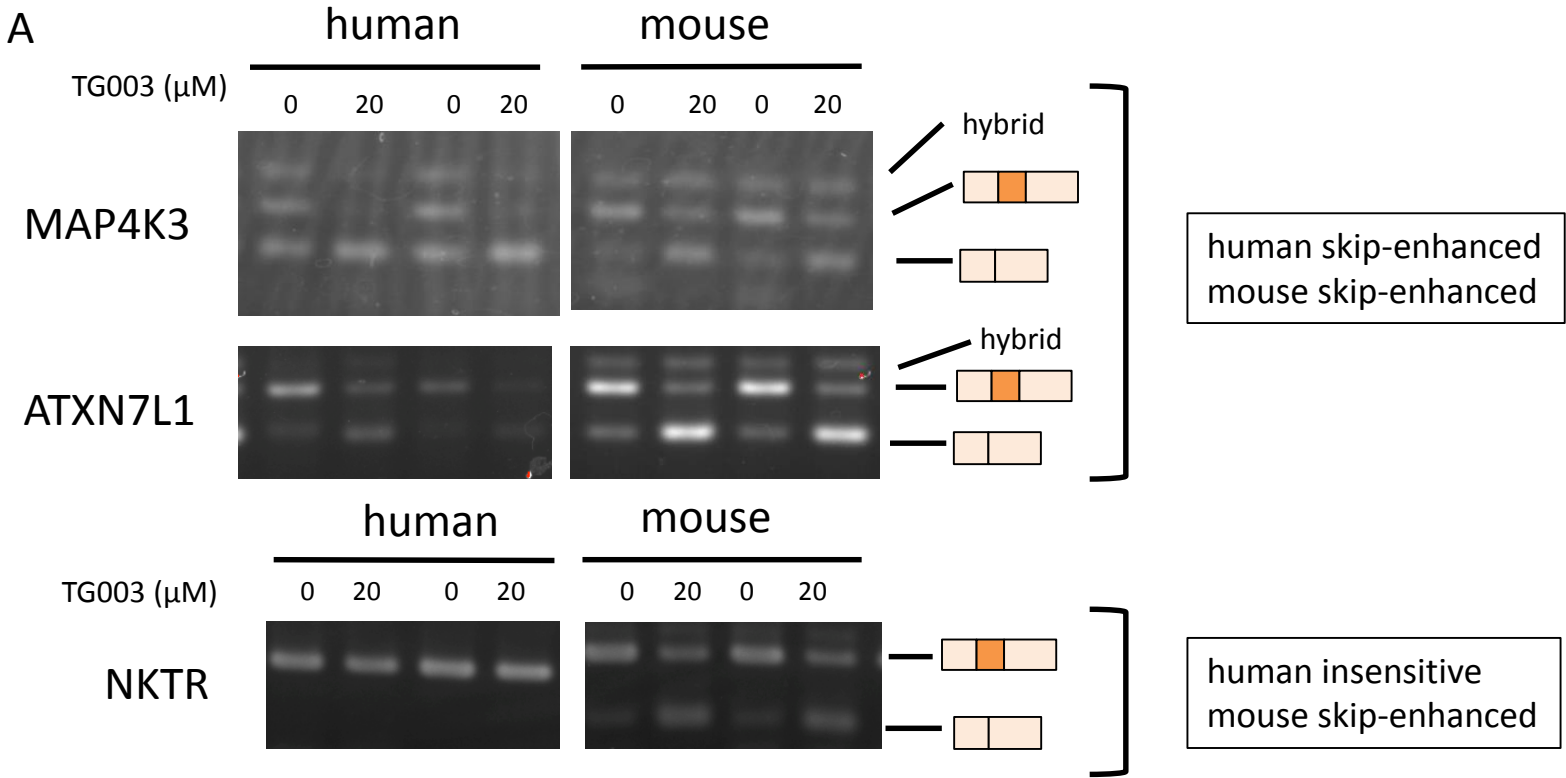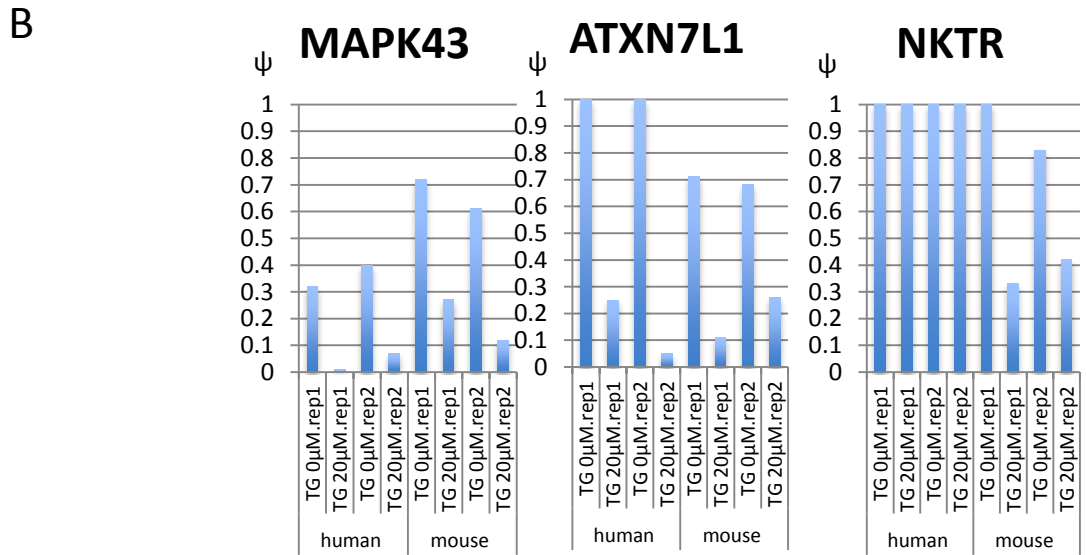

Supplement: Supplementary file 4 — Additional file 4: Figure S2. A Experimental validation of RNA-seq data of different groups.RT-PCR of two exon pairs that are both skip-enhanced by TG003 and one pair of human TG003 skip-enhanced and mouse insensitive exons. B Graphs show the RNA-seq derived ψ values. [file 12867_2015_44_MOESM4_ESM.pdf]
