# Supplementary material for: Deciphering targeting rules of splicing modulator compounds: case of TG003
Source: BMC Mol Biol. 2015 Sep 24;16:16. doi: 10.1186/s12867-015-0044-6 (PMC4580995; doi:10.1186/s12867-015-0044-6)
Supplement: Supplementary file 7 — Additional file 7: Figure S3. Alignment and upstream intron features of all 21 exon pairs. Sequence alignment of 100 nt of upstream introns and branch sequence scores, pyrimidine tract scores, and svmbp scores predicted by SVM-BP are shown. The first entry is an example and shows all the captions. [file 12867_2015_44_MOESM7_ESM.pdf]

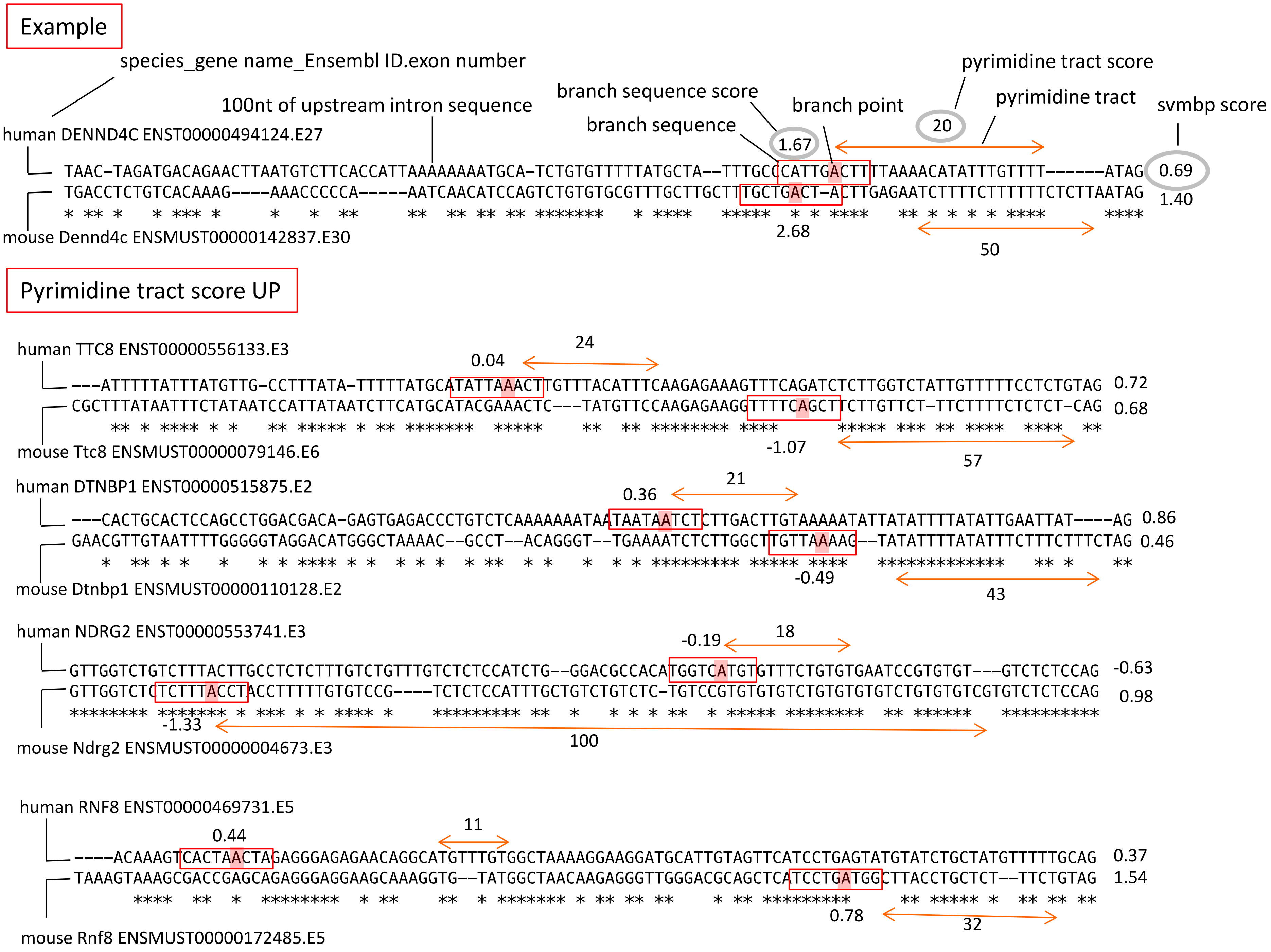

Example
species_gene name_Ensembl ID.exon number
100nt of upstream intron sequence
branch sequence score
branch sequence
branch point
pyrimidine tract score
pyrimidine tract
svmbp score
human DENND4C ENST00000494124.E27
TAAC–TAGATGACAGAACTTAATGTCTTCACCATTAAAAAAATGCA–TCTGTGTTTTTATGCTA––TTTGCCCATTGACTTTTAAAACATATTTGTTTT––––––ATAG
TGACCTCTGTCACAAAG––––AAACCCCCA–––––AATCAACATCCAGTCTGTGTGCGTTTGCTTGCTTTGCTGACT–ACTTGAGAATCTTTTCTTTTTTCTCTTAATAG
1.67
20
0.69
1.40
2.68
50
mouse Dennd4c ENSMUST00000142837.E30
Pyrimidine tract score UP
human TTC8 ENST00000556133.E3
---ATTTTTATTTATGTTG–CCTTTATA–TTTTTATGCATATTAAACTTGTTTACATTTCAAGAGAAAGTTTCAGATCTCTTGGTCTATTGTTTTTCCTCTGTAG
CGCTTTATAATTTCTATAATCCATTATAATCTTCATGCATACGAAACTC–––TATGTTCCAAGAGAAGGTTTTCAGCTTCTTGTTCT–TTCTTTTCTCTCT–CAG
24
0.04
0.72
0.68
-1.07
57
mouse Ttc8 ENSMUST00000079146.E6
human DTNBP1 ENST00000515875.E2
---CACTGCACTCCAGCCTGGACGACA–GAGTGAGACCCTGTCTCAAAAAAATAATAATAATCTCTTGACTTGTAAAAATATTATATTTTATATTGAATTAT––––AG
GAACGTTGTAATTTTGGGGGTAGGACATGGGCTAAAAC––GCCT––ACAGGGT––TGAAAATCTCTTGGCTTGTTAAAAG––TATATTTTATATTTCTTTCTTTCTAG
21
0.36
0.86
0.46
-0.49
43
mouse Dtnbp1 ENSMUST00000110128.E2
human NDRG2 ENST00000553741.E3
GTTGGTCTGTCTTTACTTGCCTCTCTTTGTCTGTTTGTCTCTCCATCTG––GGACGCCACATGGTCATGTGTTTCTGTGTGAATCCGTGTGT–––GTCTCTCCAG
GTTGGTCTCTCTTTACCTACCTTTTTGTGTCCG––––TCTCTCCATTTGCTGTCTGTCTC–TGTCCGTGTGTGTCTGTGTGTGTCTGTGTGTCGTGTCTCTCCAG
-0.19
18
-0.63
0.98
-1.33
100
mouse Ndrg2 ENSMUST00000004673.E3
human RNF8 ENST00000469731.E5
––––ACAAAGTCACTAACTAGAGGGAGAGAACAGGCATGTTTGTGGCTAAAAGGAAGGATGCATTGTAGTTCATCCTGAGTATGTATCTGCTATGTTTTTGCAG
TAAAGTAAAGCGACCGAGCAGAGGGAGGAAGCAAAGGTG––TATGGCTAACAAGAGGGTTGGGACGCAGCTCATCCTGATGGCTTACCTGCTCT––TTCTGTAG
0.44
11
0.37
1.54
0.78
32
mouse Rnf8 ENSMUST00000172485.E5

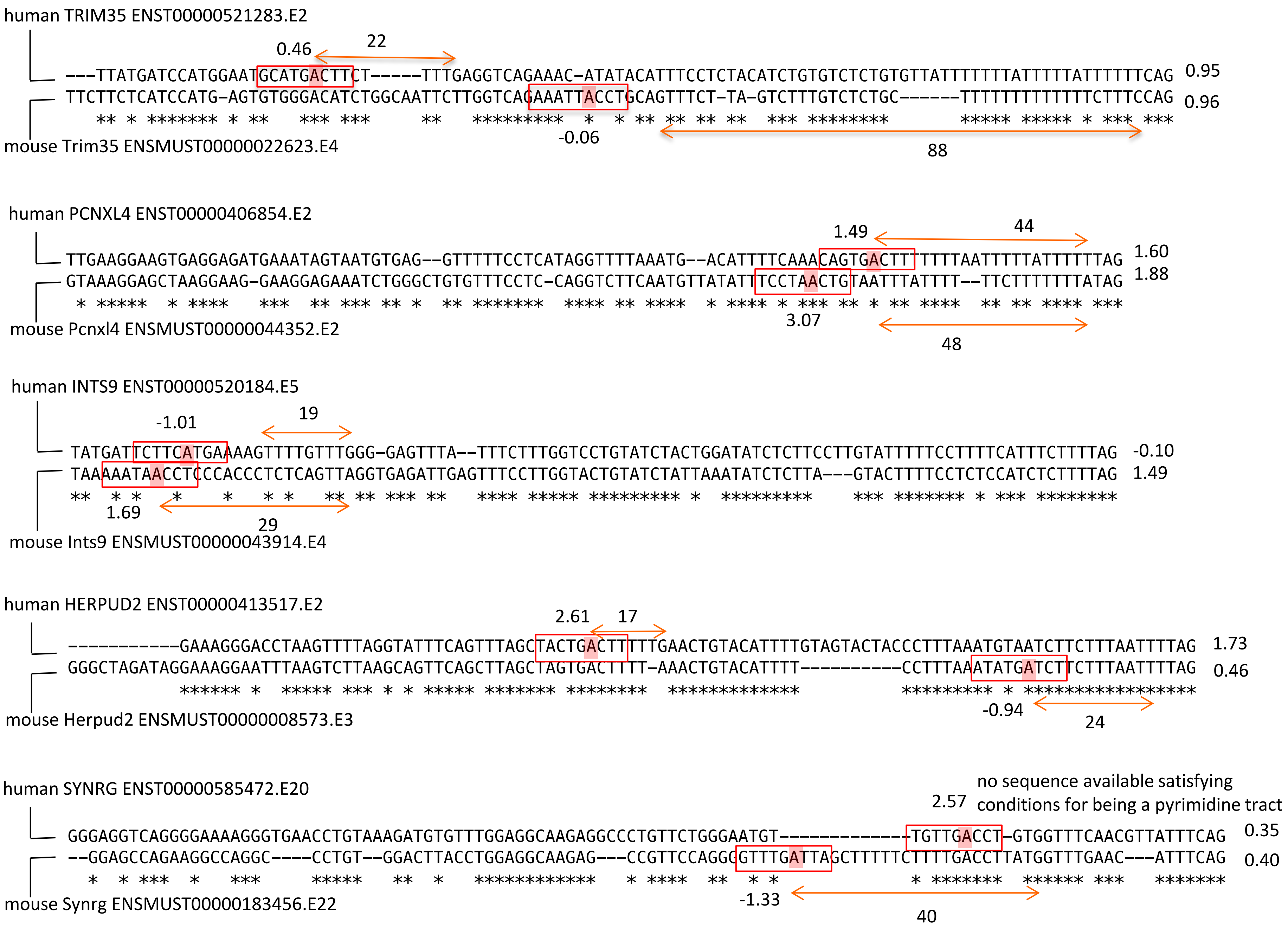

human TRIM35 ENST00000521283.E2
0.46
22
---TTATGATCCATGGAATGCATGACTTCT-----TTTGAGGTCAGAAAC-ATATACATTTCCTCTACATCTGTGTCTCTGTGTTATTTTTTTATTTTTATTTTTTCAG
0.95
TTCTTCTCATCCATG-AGTGTGGGACATCTGGCAATTCTTGGTCAGAAATTACCTGCAGTTTCT-TA-GTCTTTGTCTCTGC------TTTTTTTTTTTTTTCTTTCCAG
0.96
-0.06
88
mouse Trim35 ENSMUST00000022623.E4
human PCNXL4 ENST00000406854.E2
1.49
44
TTGAAGGAAGTGAGGAGATGAAATAGTAATGTGAG--GTTTTTCCTCATAGGTTTTAAATG--ACATTTTCAAACAGTGACTTTTTTAATTTTTATTTTTTAG
1.60
GTAAAGGAGCTAAGGAAG-GAAGGAGAAATCTGGGCTGTGTTTCCTC-CAGGTCTTCAATGTTATATTTCCTAACTGTAATTTATTTT--TTCTTTTTTTATAG
1.88
3.07
48
mouse Pcnxl4 ENSMUST00000044352.E2
human INTS9 ENST00000520184.E5
-1.01
19
TATGATTCTTCATGAAAAGTTTTGTTTGGG-GAGTTTA--TTTCTTTGGTCCTGTATCTACTGGATATCTCTTCCTTGTATTTTTCCTTTTCATTTCTTTTAG
-0.10
TAAAAATAACCTCCCACCCTCTCAGTTAGGTGAGATTGAGTTTCCTTGGTACTGTATCTATTAAATATCTCTTA---GTACTTTTCCTCTCCATCTCTTTTAG
1.49
1.69
29
mouse Ints9 ENSMUST00000043914.E4
human HERPUD2 ENST00000413517.E2
2.61
17
-----------GAAAGGGACCTAAGTTTTAGGTATTTCAGTTTAGCTACTGACTTTTTGAACTGTACATTTTGTAGTACTACCCTTTAAATGTAATCTTCTTTAATTTTAG
1.73
GGGCTAGATAGGAAAGGAATTTAAGTCTTAAGCAGTTCAGCTTAGCTAGTGACTTTT-AAACTGTACATTTT----------CCTTTAAATATGATCTTCTTTAATTTTAG
0.46
-0.94
24
mouse Herpud2 ENSMUST00000008573.E3
human SYNRG ENST00000585472.E20
no sequence available satisfying conditions for being a pyrimidine tract
2.57
GGGAGGTCAGGGGAAAAGGGTGAACCTGTAAAGATGTGTTTGGAGGCAAGAGGCCCTGTTCTGGGAATGT-------------TGTTGACCT-GTGGTTTCAACGTTATTTCAG
0.35
--GGAGCCAGAAGGCCAGGC----CCTGT--GGACTTACCTGGAGGCAAGAG---CCGTTCCAGGGTTTGATTAGCTTTTTCTTTTGACCTTATGGTTTGAAC---ATTTCAG
0.40
-1.33
40
mouse Synrg ENSMUST00000183456.E22

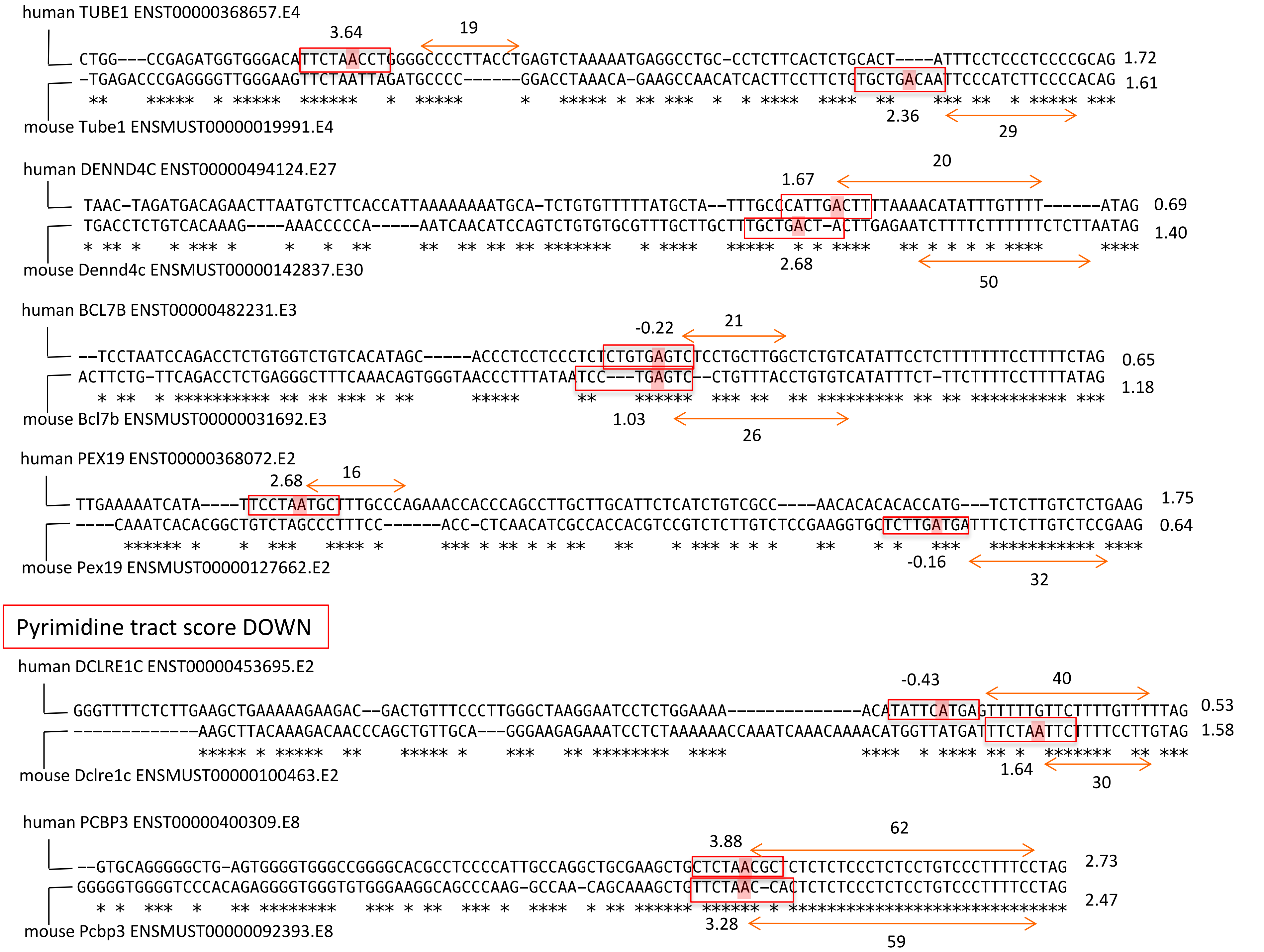

human TUBE1 ENST00000368657.E4
3.64
19
CTGG---CCGAGATGGTGGGACATTCTAACCTGGGGCCCCTTACCTGAGTCTAAAAATGAGGCCTGC-CCTCTTCACTCTGCACT----ATTTCCTCCCTCCCCGCAG 1.72
-TGAGACCCGAGGGGTTGGGAAGTTCTAATTAGATGCCCC------GGACCTAAACA-GAAGCCAACATCACTTCCTTCTGTGCTGACAATTCCCATCTTCCCCACAG 1.61
** ***** * ***** ****** * ***** * ***** * ** *** * * **** **** ** *** ** * ***** ***
2.36
29
mouse Tube1 ENSMUST00000019991.E4
human DENND4C ENST00000494124.E27
20
1.67
TAAC-TAGATGACAGAACTTAATGTCTTCACCATTAAAAAAATGCA-TCTGTGTTTTTATGCTA--TTTGCCCATTGACTTTTAAAACATATTTGTTTT------ATAG 0.69
TGACCTCTGTCACAAAG----AAACCCCCA-----AATCAACATCCAGTCTGTGTGCGTTTGCTTGCTTTGCTGACT-ACTTGAGAATCTTTTCTTTTTTCTCTTAATAG 1.40
* ** * * *** * * * ** ** ** ** ** ******* * **** ***** * * **** ** * * * * **** ****
2.68
50
mouse Dennd4c ENSMUST00000142837.E30
human BCL7B ENST00000482231.E3
-0.22
21
--TCCTAATCCAGACCTCTGTGGTCTGTCACATAGC-----ACCCTCCTCCCTCTCTGTGAGTCTCCTGCTTGGCTCTGTCATATTCCTCTTTTTTTCCTTTTCTAG 0.65
ACTTCTG-TTCAGACCTCTGAGGGCTTTCAAACAGTGGGTAACCCTTTATAATCC---TGAGTC--CTGTTTACCTGTGTCATATTTCT-TTCTTTTCCTTTTATAG 1.18
* ** * ********** ** ** *** * ** ***** ** ****** *** ** ** ********* ** ** ********** ***
1.03
26
mouse Bcl7b ENSMUST00000031692.E3
human PEX19 ENST00000368072.E2
2.68
16
TTGAAAAATCATA----TTCCTAATGCTTTGCCCAGAAACCACCCAGCCTTGCTTGCATTCTCATCTGTCGCC----AACACACACACCATG---TCTCTTGTCTCTGAAG 1.75
----CAAATCACACGGCTGTCTAGCCCTTTCC------ACC-CTCAACATCGCCACCACGTCCGTCTCTTGTCTCCGAAGGTGCTCTTGATGATTTCTCTTGTCTCCGAAG 0.64
****** * * *** **** * *** * ** * * * ** ** * *** * * * ** * * *** *********** ****
-0.16
32
mouse Pex19 ENSMUST00000127662.E2
Pyrimidine tract score DOWN
human DCLRE1C ENST00000453695.E2
-0.43
40
GGGTTTTCTCTTGAAGCTGAAAAAGAAGAC--GACTGTTTCCCTTGGGCTAAGGAATCCTCTGGAAAA--------------ACATATTCATGAGTTTTTGTTCTTTTGTTTTTAG 0.53
-------------AAGCTTACAAAGACAACCCAGCTGTTGCA---GGGAAGAGAAATCCTCTAAAAAACCAAATCAAACAAAACATGGTTATGATTTCTAATTCTTTTCCTTGTAG 1.58
***** * ***** ** ***** * *** ** ******** **** **** * **** ** * ******* ** ***
1.64
30
mouse Dclre1c ENSMUST00000100463.E2
human PCBP3 ENST00000400309.E8
62
3.88
--GTGCAGGGGGCTG-AGTGGGGTGGGCCGGGGCACGCCTCCCCATTGCCAGGCTGCGAAGCTGCTCTAACGCTCTCTCTCCCTCTCCTGTCCCTTTTCCTAG 2.73
GGGGGTGGGGTCCCACAGAGGGGTGGGTGTGGGAAGGCAGCCCAAG-GCCAA-CAGCAAAGCTGTTCTAAC-CACTCTCTCCCTCTCCTGTCCCTTTTCCTAG 2.47
* * *** * ** ******** *** * ** *** * **** * ** ****** ****** * ****************************
3.28
59
mouse Pcbp3 ENSMUST00000092393.E8

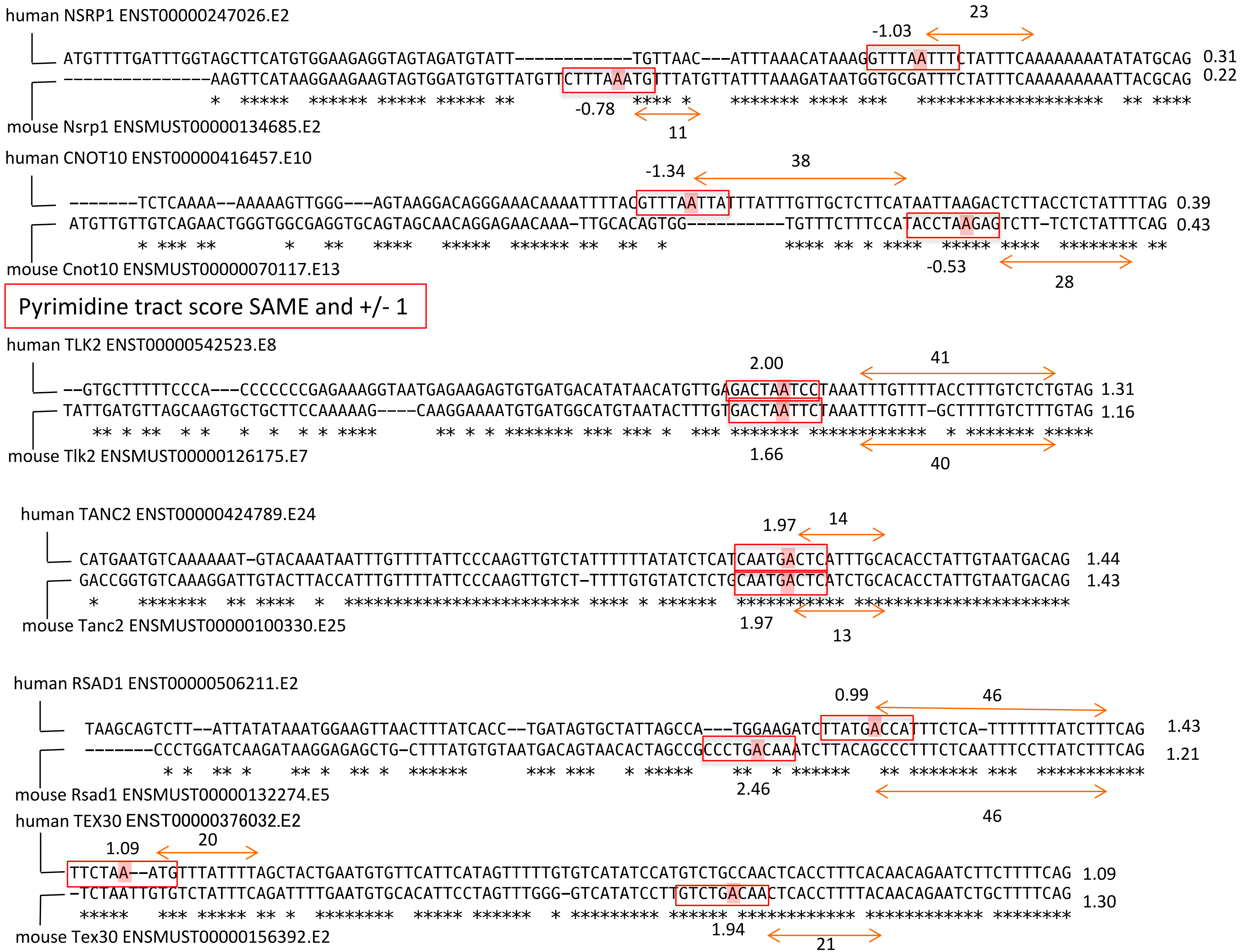
human NSRP1 ENST00000247026.E2
23
-1.03
ATGTTTTGATTTGGTAGCTTCATGTGGAAGAGGTAGTAGATGTATT------------TGTTAAC---ATTTAAACATAAAGGTTTAATTTCTATTTCAAAAAAAATATATGCAG 0.31
---------------AAGTTCATAAGGAAGAAGTAGTGGATGTGTTATGTTCTTTAAATGTTTATGTTATTTAAAGATAATGGTGCGATTTCTATTTCAAAAAAAAATTACGCAG 0.22
* ***** ****** ***** ***** ** **** * ******* **** *** ****************** ** ****
-0.78
11
mouse Nsrp1 ENSMUST00000134685.E2
human CNOT10 ENST00000416457.E10
-1.34
38
-------TCTCAAAA--AAAAAGTTGGG---AGTAAGGACAGGGAAACAAAATTTTACGTTTAATTATTTATTTGTTGCTCTTCATAATTAAGACTCTTACCTCTATTTTAG 0.39
ATGTTGTTGTCAGAACTGGGTGGCGAGGTGCAGTAGCAACAGGAGAACAAA-TTGCACAGTGG----------TGTTTCTTTCCATACCTAAGAGTCTT-TCTCTATTTCAG 0.43
* *** ** * ** **** ***** ****** ** ** * **** ** * **** ***** **** ******** **
mouse Cnot10 ENSMUST00000070117.E13
-0.53
28
Pyrimidine tract score SAME and +/- 1
human TLK2 ENST00000542523.E8
2.00
41
--GTGCTTTTTCCCA---CCCCCCCGAGAAAGGTAATGAGAAGAGTGTGATGACATATAACATGTTGAGACTAATCCTAAATTTGTTTTACCTTTGTCTCTGTAG 1.31
TATTGATGTTAGCAAGTGCTGCTTCCAAAAAG----CAAGGAAAATGTGATGGCATGTAATACTTTGTGACTAATTCTAAATTTGTTT-GCTTTTGTCTTTGTAG 1.16
** * ** * * * * * * **** ** * * ******* *** *** * *** ******* ************ * ******* *****
mouse Tlk2 ENSMUST00000126175.E7
1.66
40
human TANC2 ENST00000424789.E24
1.97
14
CATGAATGTCAAAAAAT-GTACAAATAATTTGTTTTATTCCCAAGTTGTCTATTTTTTATATCTCATCAATGACTCATTTGCACACCTATTGTAATGACAG 1.44
GACCGGTGTCAAAGGATTGTACTTACCATTTGTTTTATTCCCAAGTTGTCT-TTTTGTGTATCTCTGCAATGACTCATCTGCACACCTATTGTAATGACAG 1.43
* ******* ** **** * *********************** **** * ****** *********** *********************
mouse Tanc2 ENSMUST00000100330.E25
1.97
13
human RSAD1 ENST00000506211.E2
0.99
46
TAAGCAGTCTT--ATTATATAAATGGAAGTTAACTTTATCACC--TGATAGTGCTATTAGCCA---TGGAAGATCTTATGACCATTTCTCA-TTTTTTTATCTTTCAG 1.43
-------CCCTGGATCAAGATAAGGAGAGCTG-CTTTATGTGTAATGACAGTAACACTAGCCGCCCTGACAAATCTTACAGCCCTTTCTCAATTTCCTTATCTTTCAG 1.21
* * ** * ** * ** * ****** *** *** * ***** ** * ****** ** ******* *** ***********
mouse Rsad1 ENSMUST00000132274.E5
2.46
46
human TEX30 ENST00000376032.E2
1.09
20
TTCTAA--ATGTTTATTTTAGCTACTGAATGTGTTCATTCATAGTTTTTGTGTCATATCCATGTCTGCCAACTCACCTTTCACAACAGAATCTTCTTTTCAG 1.09
-TCTAATTGTGTCTATTTCAGATTTTGAATGTGCACATTCCTAGTTTGGG-GTCATATCCTTGTCTGACAACTCACCTTTTACAACAGAATCTGCTTTTCAG 1.30
***** *** ***** ** * ******** ***** ****** * ********* ****** ************ ************ ********
1.94
21
mouse Tex30 ENSMUST00000156392.E2
