# Supplementary material for: Deciphering targeting rules of splicing modulator compounds: case of TG003
Source: BMC Mol Biol. 2015 Sep 24;16:16. doi: 10.1186/s12867-015-0044-6 (PMC4580995; doi:10.1186/s12867-015-0044-6)
Supplement: Supplementary file 8 — Additional file 8: Figure S4. Boxplots of homologous exon pairs that both respond to TG003. Boxplots with additional data pair set (5-6 both skip-enhanced, n = 9 as in Figure 3B red) to Figure 5. Significance is shown only for Wilcoxon rank sum test performed with 5 or 6. Median is shown by bold horizontal line and boxes are drawn between the third quartile (top of box) and first quartile (bottom of box). Outliers are either 3 times interquartile range or more above the third quartile, or 3 times interquartile range or more below the first quartile and are not shown in the plot. The maximum and minimum are shown with the whiskers. [file 12867_2015_44_MOESM8_ESM.pdf]

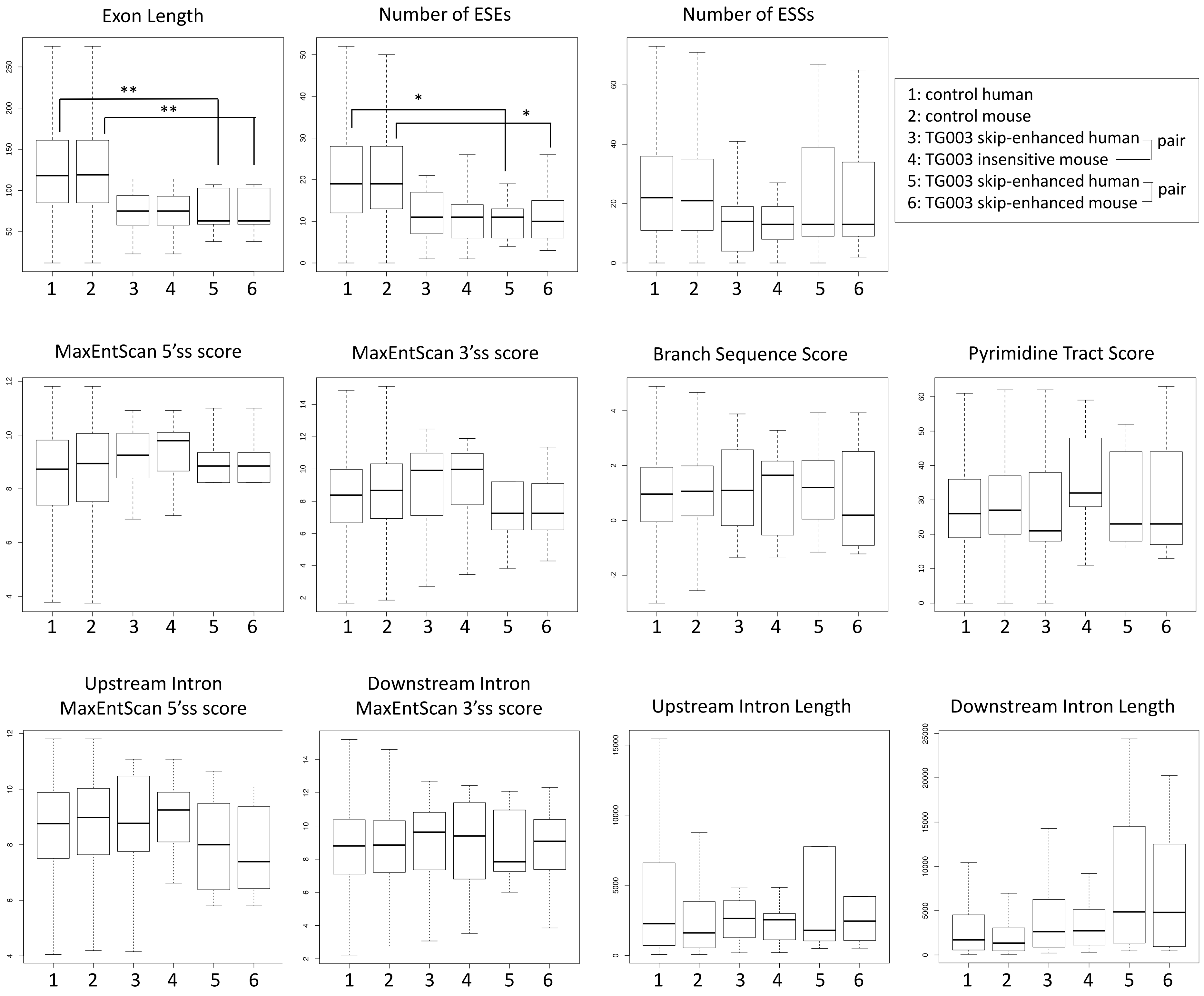

Exon Length
Number of ESEs
Number of ESSs
1: control human
2: control mouse
3: TG003 skip-enhanced human
4: TG003 insensitive mouse
pair
5: TG003 skip-enhanced human
6: TG003 skip-enhanced mouse
pair
MaxEntScan 5'ss score
MaxEntScan 3'ss score
Branch Sequence Score
Pyrimidine Tract Score
Upstream Intron MaxEntScan 5'ss score
Downstream Intron MaxEntScan 3'ss score
Upstream Intron Length
Downstream Intron Length
1 2 3 4 5 6
**
*
