# Supplementary material for: Deciphering targeting rules of splicing modulator compounds: case of TG003
Source: BMC Mol Biol. 2015 Sep 24;16:16. doi: 10.1186/s12867-015-0044-6 (PMC4580995; doi:10.1186/s12867-015-0044-6)
Supplement: Supplementary file 12 — Additional file 12: Figure S5. Methods to identify TG003 sensitive exons and sequence features of interest.A. The positions of the genomic coordinates used to identify exon-skipping events are shown in red with numbered arrows. Boxes represent exons and lines represent introns. B. Overview of the calculation of percent spliced in (ψ) values. Red lines represent RNA-seq reads mapped to the genome. Circled numbers represents the number of reads mapped to the exon junction right below it. C. The positions and length of the sequences used as inputs to the respective scoring systems. [file 12867_2015_44_MOESM12_ESM.pdf]

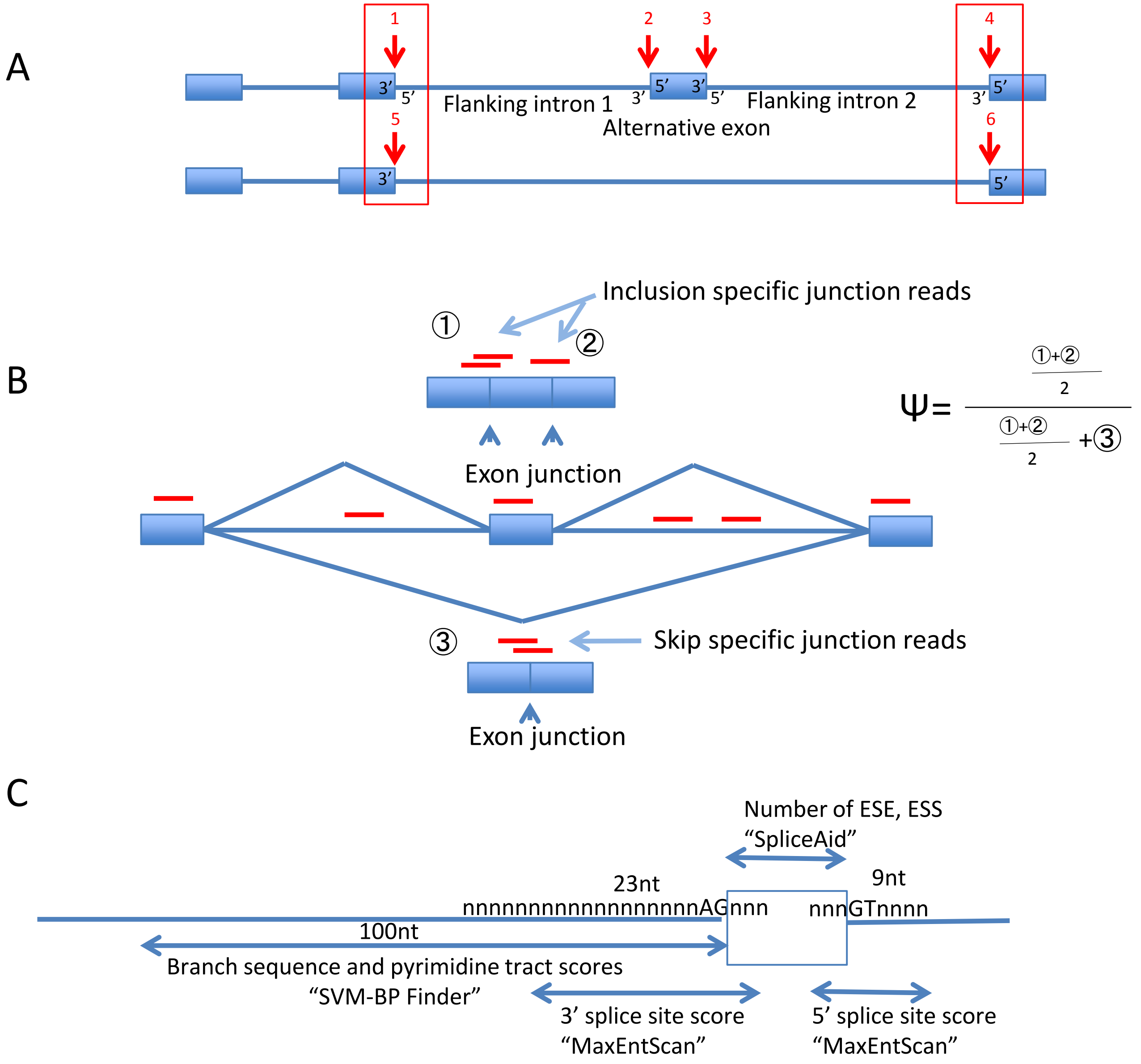
A
1
2
3
4
3’
5’
Flanking intron 1
3’
5’
3’
5’
Flanking intron 2
3’
5’
Alternative exon
5
6
3’
5’
B
Inclusion specific junction reads
①
②
Exon junction
$\Psi = \frac{\frac{①+②}{2}}{\frac{①+②}{2}+③}$
③
Skip specific junction reads
Exon junction
C
Number of ESE, ESS
“SpliceAid”
23nt
9nt
nnnnnnnnnnnnnnnnnnAGnnn
nnnGTnnnnn
100nt
Branch sequence and pyrimidine tract scores
“SVM-BP Finder”
3’ splice site score
“MaxEntScan”
5’ splice site score
“MaxEntScan”
